# Human Platelet-Rich Plasma Regulates Canine Mesenchymal Stem Cell Migration through Aquaporins

**DOI:** 10.1155/2023/8344259

**Published:** 2023-05-15

**Authors:** Alessia Parascandolo, Michele Francesco Di Tolla, Domenico Liguoro, Manuela Lecce, Saverio Misso, Fabiana Micieli, Maria Rosaria Ambrosio, Serena Cabaro, Francesco Beguinot, Alessandra Pelagalli, Vittoria D'Esposito, Pietro Formisano

**Affiliations:** ^1^Department of Translational Medical Sciences, University of Naples “Federico II”, Via Pansini 5, 80131 Naples, Italy; ^2^URT “Genomic of Diabetes”, Institute for Experimental Endocrinology and Oncology “G. Salvatore”, National Research Council (IEOS-CNR), Via Pansini 5, 80131 Naples, Italy; ^3^Unit of Transfusion Medicine, Azienda Sanitaria Locale Caserta, Caserta, Italy; ^4^Department of Veterinary Medicine and Animal Productions, University of Napoli Federico II, 80137 Naples, Italy; ^5^Department of Advanced Biomedical Sciences, University of Napoli Federico II, 80131 Naples, Italy; ^6^Institute of Biostructures and Bioimages, National Research Council, 80145 Naples, Italy

## Abstract

Platelet products are commonly used in regenerative medicine due to their effects on the acceleration and promotion of wound healing, reduction of bleeding, synthesis of new connective tissue, and revascularization. Furthermore, a novel approach for the treatment of damaged tissues, following trauma or other pathological damages, is represented by the use of mesenchymal stem cells (MSCs). In dogs, both platelet-rich plasma (PRP) and MSCs have been suggested to be promising options for subacute skin wounds. However, the collection of canine PRP is not always feasible. In this study, we investigated the effect of human PRP (hPRP) on canine MSCs (cMSCs). We isolated cMSCs and observed that hPRP did not modify the expression levels of the primary class of major histocompatibility complex genes. However, hPRP was able to increase cMSC viability and migration by at least 1.5-fold. hPRP treatment enhanced both Aquaporin (AQP) 1 and AQP5 protein levels, and their inhibition by tetraethylammonium chloride led to a reduction of PRP-induced migration of cMSCs. In conclusion, we have provided evidence that hPRP supports cMSC survival and may promote cell migration, at least through AQP activation. Thus, hPRP may be useful in canine tissue regeneration and repair, placing as a promising tool for veterinary therapeutic approaches.

## 1. Introduction

Platelets represent a relevant source of growth factors involved in the regulation of growth, differentiation, and metabolism of cells. For instance, platelet-rich plasma (PRP) contains several proteins such as platelet-derived growth factors (PDGF), transforming growth factors *β* (TGF-*β*), insulin-like growth factors (IGF-1), fibroblast growth factors (FGF), fibrinogen, fibronectin, and cytokines that play an important role during tissue repair and regeneration [1–3].

Mesenchymal stem/stromal cells (MSCs) are multipotent cells characterized by a high proliferation rate, multilineage differentiation capability, and large paracrine activity. MSCs are recruited in damaged tissues and manage several steps of the wound healing and regeneration process, including the control of inflammation, neovascularization and reepithelialization, fibroblast activation, and production of collagen [4]. MSCs are largely used in clinical applications, as a treatment of damaged tissues, following trauma or other pathological damages [5]. Bone marrow represents the most widely used source of MSCs; however, subcutaneous and visceral adipose tissue represent an equally valid alternative source [6].

Recent *in vitro* and *in vivo* studies highlighted the ability of PRP to support human mesenchymal stem cell (MSC) growth and secretory functions, even in a diabetic-like environment [7–9]. PRP and MSCs exert a synergistic activity due to their immunomodulatory functions and to the capacity of PRP to be a suitable scaffold for cell-based therapies [7, 10, 11]. In particular, adipose-derived MSCs (adMSCs) have the ability to stimulate repair and regeneration of the skin in injured tissue while PRP, due to its high content of growth factors, provides the microenvironmental stimuli to enhance MSC proliferation and differentiation [12]. Interestingly, in diabetic foot ulcers, the combined use of adMSC and PRP showed reepithelialization and granulation tissue formation in the damaged area, leading to accelerate postoperative wound healing [13].

In the veterinary field, platelet concentrates, including PRP, are used in various diseases characterized by impaired repair process of damaged tissue [14]. As in humans, positive results have been shown, particularly for the treatments of canine nonhealing wounds, either in acute wound experimental models or in the clinical practice. In dogs, different studies have reported that PRP accelerates and stimulates the healing of acute wounds and promotes the repair of impaired chronic wounds [15]. PRP and MSC could be a valid alternative therapy to promote cutaneous wound healing in subacute skin wounds when traditional surgery cannot be performed in dogs [16]. Indeed, the combined use of PRP and MSCs may also overcome the limiting role played by inflammation on MSC functions [7, 10, 11, 16].

Thus, PRP is a valuable, readily available, and cost-effective therapy for wound healing in dogs. However, blood from drowning is not always easily available. Canine PRP preparation systems require about 40 ml of blood to obtain about 6 ml of PRP product, and often more PRP applications are recommended with larger PRP amounts [15, 17]. This could be a problem for dogs, especially for small sized dogs. Moreover, there are situations where animals, as humans, have physiologic conditions compromised and may not be possible to collect their own blood in enough amounts for autologous PRP. In those situations, heterologous PRP (obtained from other species) may be useful, as in rabbits, where canine PRP contributed to wound healing similar to homologous PRP, with no adverse effects [18, 19]. Thus, it would also be plausible to use PRP of human origin.

Here, we have isolated canine MSCs (cMSCs) and evaluated the human PRP and cMSCs interactions focusing on cell growth, immunogenicity, migration and pathways activation.

## 2. Material and Methods

### 2.1. Canine Mesenchymal Stem Cell Isolation and Growth

Subcutaneous adipose tissue biopsies were obtained from *N* = 2 medium-sized dogs: a Breton female dog two years old upon hysterectomy and a Golden Retriever male dog 9 months old upon orchiopexy. The procedure was approved by the Veterinary Service Center of the University of Naples Federico II, and the owners gave verbal consent to perform surgical procedures. Adipose tissue samples from both dogs were subjected to mechanical cutting and enzymatically digested with type VIII collagenase (1 mg/ml from Sigma-Aldrich, St. Louis, MO, USA; cat n. C2139) at 37°C for 1 h to isolate canine MSC (cMSC) [7, 20]. cMSCs were cultured in DMEM (Dulbecco's modified Eagle's Medium)-F12 (1 : 1) plus 20% Fetal Bovine Serum (FBS) medium supplemented with 2-mM glutamine, 100-unit/ml penicillin, and 100-unit/ml streptomycin (Lonza Group Ltd., Basel, Switzerland). For all experiments, cMSCs were used between the 4th and the 6th passages. Both cell isolates were used for each experiment. To evaluate cMSC growth, 1.5x10^4^ cells were seeded in 12-well culture plates and counted upon 24-, 48-, and 72 hours with the TC10™ automated cell counter (Bio-Rad, Hercules, CA, USA) according to the manufacturer's protocol. Osteogenic differentiation was performed as previously described [7]. The presence of mineralization foci was demonstrated by Alizarin Red S staining (Sigma-Aldrich, St. Louis, MO, USA). Adipogenic differentiation was achieved as described in [21]. Differentiated cells were photographed by the Olimpus DP20 microscope digital camera system (Olympus Corporation, Tokyo, Japan).

### 2.2. Flow Cytometry Analysis

Cytofluorimetric analysis was conducted to analyze the expression of mesenchymal markers on both c-MSC isolates. 2 × 10^5^ cells were incubated with FITC-conjugated anti-CD-90 (BD Biosciences, San Jose, CA, USA, cat n. 555595), PE-conjugated anti-CD-44 (Miltenyi Biotec, Bergisch Gladbach, Germany, cat n. 130-102-606), PE-conjugated anti-CD-105 (Miltenyi Biotec, Bergisch Gladbach, Germany, cat n. 130-118-174), and APC-H7-conjugated anti-CD-45 (BD Biosciences, San Jose, CA, USA, cat n. 641399) as well as dye/isotype-matched antibodies in the dark environment for 30 min at 4°C. All samples were processed by a BD LSR Fortessa (BD Biosciences, San Jose, CA, USA) and analyzed with BD FACS Diva Software. For each sample, 10^4^ events were acquired in all analyses.

### 2.3. Human Platelet Rich Plasma (PRP) Preparation

Human platelet-rich plasma (hPRP) was obtained from the plasma of healthy blood donors, as previously described [7, 14], and stored at -20°C. For platelet gel preparations, PRP samples were incubated with calcium gluconate (Galenica senese, -10 mg/ml final concentration) for 1 h at 37°C to allow clot formation (hPRP gel) [8, 14]. Conditioned media (hPRP-CM) were obtained by incubating hPRP gel for 48 h with serum-free DMEM-F12 (1 : 1) and 0.25% bovine serum albumin (BSA) (10% vol/vol in DMEM F12-BSA). After the incubation, the medium was collected, centrifuged at 14,000 g to remove cellular debris, and applied on to cMSCs. hPRP-CM holds all factors released by hPRP gel in 48 h, including cytokines, chemokines, and growth factors [3]. The use of hPRP-CM overcomes some disadvantages of hPRP gel such as the direct contact of the gel with the cells, which may cause the detachment of the cells from the plates. Thus, all experiments were performed using hPRP-CM. Migration assay was carried out either with hPRP-CM or with hPRP gel added in the lower chamber of the transwell system, not in direct contact with the cell layer.

### 2.4. Proliferation Assays

For the growth curve, 3 × 10^3^ cMSCs were seeded in 96-well plates in DMEM F12 (1 : 1) with 20% FBS. The following day, cells were starved in serum-free DMEM F12 (1 : 1) 0.25% BSA for 16 h and incubated with hPRP-CM for 48,72, 96, and 120 hours. As a control, cells were incubated with DMEM F12 (1 : 1) with 0.25% BSA or with 20% FBS (standard growth conditions). Cell proliferation was analyzed with the Cell Titer 96 Aqueous One Solution Cell Proliferation assay, a colorimetric method for determining the number of viable cells in proliferation assays (#G3580, Promega Corporation, Madison, USA), according to the manufacturer's instructions.

To evaluate the effect of tetraethylammonium chloride (TEAC-Sigma T2265, Saint Louis, USA) on cMSC viability, 5 × 10^3^ cells were seeded in 96-well plates in a DMEM F12 (1 : 1) with 20% FBS. The following day, cells were treated with three different concentrations of TEAC (10 *μ*M, 50 *μ*M, and 100 *μ*M) in DMEM F12 (1 : 1) with 0.25% BSA medium for 48 h. Cell viability was evaluated by the sulforhodamine B assay (Sigma-Aldrich, St. Louis, MO, USA), as previously described [20, 22].

### 2.5. Migration Assay

Cell migration was performed using 8 *μ*m-pore polycarbonate membranes (Costar, Cambridge, MA, USA). 5 × 10^4^ cMSCs were loaded on the upper chamber of the transwell system and hPRP-CM or hPRP gel in DMEM F12 (1 : 1). 0.25% BSA (10% vol/vol in DMEM F12-BSA) was added to the lower chamber. Cells were allowed to migrate into the lower chamber at 37°C in a 5% CO_2_ atmosphere saturated with H_2_O for 48 h. At the end of incubation, cells that had migrated to the lower side of the filter were fixed with 11% glutaraldehyde (G6257-1 L, Sigma-Aldrich) for 15 min at room temperature, washed three times with phosphate buffered saline (PBS), and stained with 0.1% crystal violet (Sigma-Aldrich, St. Louis, MO, USA) and 20% methanol for 20 min at room temperature. After three PBS washes and complete drying at room temperature, the crystal violet was solubilized by immersing the filters in 0.1 M sodium citrate. The concentration of the solubilized crystal violet was evaluated as absorbance at 540 nm at the GloMax® Discover Microplate Reader (Promega Corporation, Madison, USA).

### 2.6. Immunoblot Procedure

Protein lysates were prepared according to standard procedures [23]. Briefly, to obtain protein lysates, cMSCs were harvested in JS lysis buffer (HEPES 50 mM, MgCl_2_ 1.5 mM, NaCl 150 mM, EGTA 5 mM, 1% Triton, and 1% Glycerol) and centrifuged at 14,000 rpm for 30 min. Protein quantification was determined at a Beckman Coulter spectrophotometer (DU®530, Life Science UV/VIS) using the Breadford Assay (Bio-Rad Protein Assay Dye #5000006). 20 *μ*g of cell lysates were separated by SDS-polyacrylamide gel electrophoresis and blotted on Immobilon-P membranes (Millipore, Billerica, MA, USA). Membranes were blocked for 1 h in Tris Buffer Saline (TBS) (10 mM Tris-HCl, pH 7.4, and 140 mM NaCl) containing 3% (*w*/*v*) BSA and then incubated with the following antibodies: anti-AQP1 (ab15080, rabbit polyclonal, Abcam), anti-AQP5 (STJ111968, rabbit polyclonal, St John's Laboratory), anti-p-ERK1/2 Thr202/Tyr204 (#9101, rabbit polyclonal, cell signaling), anti-p-AKT/PKB S473 (sc-7985-R, rabbit polyclonal, SantaCruz), anti-p-FAK S910 (sc-16666, goat polyclonal, SantaCruz), and anti-Vinculin (sc-73614, mouse monoclonal, SantaCruz). Secondary antibodies used were anti-rabbit (170-6515, Bio-Rad), anti-mouse (170-6516, Bio-Rad), and anti-goat (sc-2020, SantaCruz). Antigen-antibody complexes were revealed by enhanced chemiluminescent detection kit (LiteAblot®PLUS, EuroClone SpA, Milan, Italy) according to the manufacturer's instruction. Densitometric analyses have been performed on autoradiographs with ImageJ software.

### 2.7. RNA Isolation and Quantitative Real-Time PCR (Q-RT-PCR)

Total RNA was isolated using TRIzol solution (Life Technologies, Carlsbad, CA, USA) according to the manufacturer's instructions. 500 *μ*g of RNA from each sample was reverse transcribed with SuperScript II Reverse Transcriptase with oligo dT primers (Invitrogen, Carlsbad, CA, USA). Quantitative real-time PCR was carried out with iTaq Universal SYBR Green Supermix (Biorad, Hercules, CA, USA), as previously described [24]. All reactions were performed in duplicate, and glyceraldehyde 3-phosphate dehydrogenase (GAPDH) was used as the internal reference. Primer sequences for Dog Leukocyte Antigen 12 (DLA12), DLA64, and GAPDH were as follows: DLA12 forward CAACCTCTGTGTCCTGGGTC and reverse TACCTGGAGATGGGGAAGGA; DLA64 forward TGGCGGGTCAGGTAGATTTT and reverse GATTACCTGGCCCTGGACG; and GAPDH forward ACAGTCAAGGCTGAGAACGG and reverse CCACAACATACTCAGCACCAGC. Fold changes were calculated with 2^−*ΔΔ*Ct^ formula.

### 2.8. Statistical Analysis

Statistical analyses were performed with GraphPad Prism 7.0 software (GraphPad Software Inc., La Jolla, CA). Results are presented as the mean ± standard deviation (SD). For comparisons between 2 groups, a two-tailed *t*-test for independent samples was used. Multiple comparisons among the three groups were made using the ANOVA test with Tukey correction. *p* value of < 0.05 were considered statistically significant.

## 3. Results

### 3.1. Isolation and Characterization of Canine MSCs

**Figure 1:**
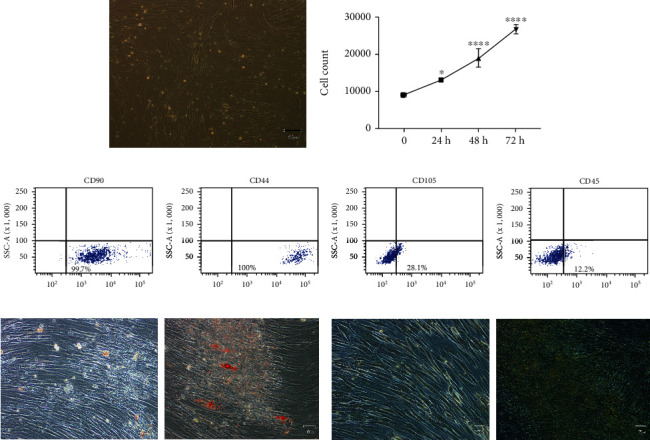
Isolation and characterization of cMSCs. (a) cMSCs were isolated from subcutaneous adipose tissue and plated (magnification 10x). (b) cMSCs were grown in DMEM-F12 20% FBS and counted at 24 h, 48 h, and 72 h. The data obtained represent the mean ± SD of three independent experiments. The statistical analysis was calculated by the one-way ANOVA test. Asterisks denote statistical significance versus time point zero (^∗^*p* < 0.05, ^∗∗∗∗^*p* ≤ 0.0001). (c) Representative quadrant dot plots from FACS analysis of cMSCs stained for CD90, CD44, CD105, and CD45 antigens. (d) Representative microscopic images (magnification 20x) from Alizarin Red staining (ARS) for calcium accumulation detection in undifferentiated and osteoblast-like differentiated cells. (e) Representative microscopic images (magnification 20x) of undifferentiated and adipocyte-differentiated cells.

Canine MSCs (cMSCs) were isolated from subcutaneous adipose tissue, plated, and grown as described in Materials and Methods. Cells formed a monolayer of fibroblast-like-shaped cells (Figure 1(a)) and displayed an increase in cell number of about 30% every 24 h (Figure 1(b)). Moreover, cells stained positively for mesenchymal progenitor cell surface antigens CD90, CD44, and CD105 and negatively for the hematopoietic marker CD45 (Figure 1(c)).

To further demonstrate the stem features of cMSCs, cells were induced to differentiate osteoblast-like cells and in adipocytes. In the presence of osteogenic stimuli, cMSCs displayed intracellular calcium accumulation (Figure 1(d)), while when cultured with an adipogenic induction mix, cMSCs showed lipid droplets (Figure 1(e)).

### 3.2. Human PRP (hPRP) Promotes cMSC Viability and Migration

**Figure 2:**
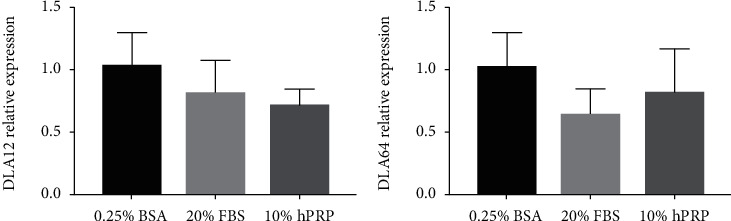
hPRP effect on MHC-I genes. mRNA levels of DLA12 and DLA64 were determined by qPCR analysis of total RNA isolated from cMSCs treated with 10% hPRP for 48 h. The average expression value of DLA12 (a) and DLA64 (b) in cMSCs treated with DMEM-F12 0.25% BSA (control cells) was used as a reference sample and GAPDH as a housekeeping gene.

We focused on the effects of hPRP-released factors on cMSCs. First, we analyzed expression of the primary class of major histocompatibility complex I (MHC-I) genes, DLA12 and DLA64 by real-time PCR in cMSCs treated with 10% hPRP-CM for 48 h. Cells did not show increased DLA12 and DLA64 mRNA levels compared to cells grown in serum-free medium (0.25% BSA) or in standard growth condition (20% FBS) (Figure 2).

**Figure 3:**
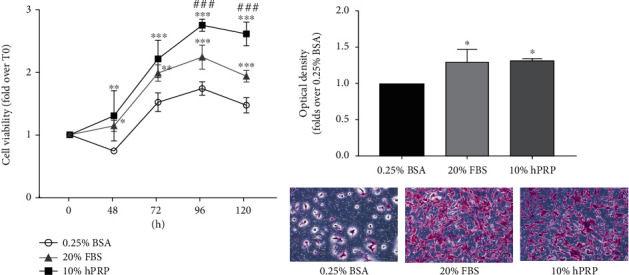
hPRP effect on cMSC proliferation and migration. (a) cMSCs were treated with 10% hPRP-CM (obtained in 0.25% BSA medium, as described in Material and Methods) for 48, 72, 96, and 120 hours. As a control, cells were seeded in DMEM F12 (1 : 1) with 0.25% BSA or with 20% FBS (standard growth conditions). Cell viability was analyzed by the MTT assay. (b) cMSCs were serum-starved for 18 h and then seeded in the upper chamber of a transwell culture system. hPRP gel (10% vol/vol in DMEM F12 with 0.25% BSA) was added in the lower chamber in the presence of 0.25% BSA medium for 48 h. As a control, DMEM F12 (1 : 1) without serum supplementation (0.25% BSA) or with 20% FBS (standard growth conditions) was added to the lower compartment. Cells that migrated across the filter were determined by crystal violet staining, as described in Materials and Methods. Bars show the fold-over control represented by cells in 0.25% BSA. Before crystal violet elution, cells were photographed (magnification 10x). (a, b) The data represent the mean ± SD of three independent experiments. ^∗^ denotes statistical significance versus cells in 0.25% BSA medium (^∗^*p* < 0.05; ^∗∗^*p* < 0.01; ^∗∗∗^*p* < 0.001); # denotes statistical significance versus cells in 20% FBS medium (###*p* < 0.001).

Thus, we investigated the effects of hPRP on cMSC functions. As shown in Figure 3(a), 10% hPRP induced a significant increase in cMSC proliferation, from 48 h to 5 days of treatment, compared to control cells (cells in 0.25% BSA medium). Upon 48 and 72 hours, hPRP effect was similar to that obtained in standard growth condition (DMEM F12, 20% FBS). Interestingly, upon 96- and 120-hour treatments, hPRP induced an increase of cMSC proliferation significantly higher also compared to 20% FBS medium (Figure 3(a)).

Next, we studied the ability of hPRP to promote cMSC migration. Thus, we performed a transwell migration assay by filling the bottom of the transwell dish with activated 10% hPRP-gel in serum-free medium and seeding cMSCs into the top chamber. After 48 h, cells that migrated across the filter were detected, photographed, and quantified. As shown in Figure 3(b), the presence of hPRP in 0.25% BSA medium increased cMSC migration by about 1.5-fold compared to cells in 0.25% BSA medium, a migration rate comparable to that achieved in standard growth condition medium (20% FBS medium). In addition, hPRP CM enhanced cMSC migration in a similar way to hPRP gel (Supplementary Figure 1), indicating a role for hPRP-released factors in regulating the migratory properties of cMSCs.

Moreover, immunoblot experiments revealed that 10% hPRP increased p-ERK, p-AKT/PKB, and p-FAK levels compared to control cells (Supplementary Figure 2).

Thereby, our data suggest that hPRP-released factors are able to sustain cMSC viability and migration and activate multiple signaling pathways.

### 3.3. AQPs Are Involved in cMSC Migration

**Figure 4:**
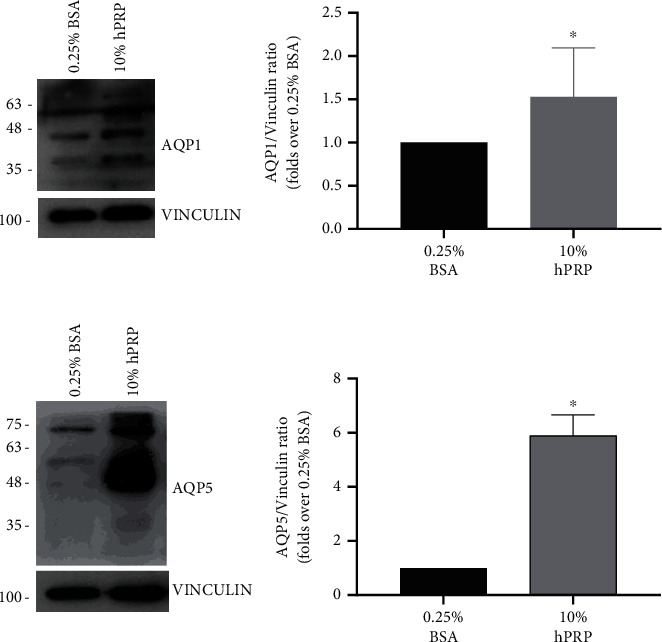
hPRP effect on cMSC AQPs. cMSCs were treated with 10% hPRP-conditioned medium for 24 h. Cell lysates (20 *μ*g protein/sample) were blotted with AQP1 (a) and AQP5 (b) antibodies. To ensure equal protein transfer, membranes were blotted with vinculin antibodies. Filters were revealed by enhanced chemiluminescence (ECL) and autoradiography. The autoradiographs shown are representative of three independent experiments. Densitometric analyses have been performed on autoradiographs. Bars show the ratio between AQPs and vinculin protein levels as a fold-over control (cells in 0.25% BSA). The data were analyzed using the Mann–Whitney test (^∗^*p* < 0.05).

Recent evidence suggested a crucial role for aquaporin (AQP) water channels in the regulation and support of cellular migration ([25]; Hyun Jun [26–28]). However, the involvement of AQPs in cMSC migration, as well as their induction by hPRP, have not been investigated yet. Here, we evaluated AQP1 and AQP5 protein levels in cMSCs treated with 10% hPRP. Interestingly, hPRP stimulation led to a significant increase in both AQP1 (Figure 4(a)), detected as a double band of 40-50 kDa [29], and AQP5 (Figure 4(b)) protein levels in cMSCs, compared to control cells.

**Figure 5:**
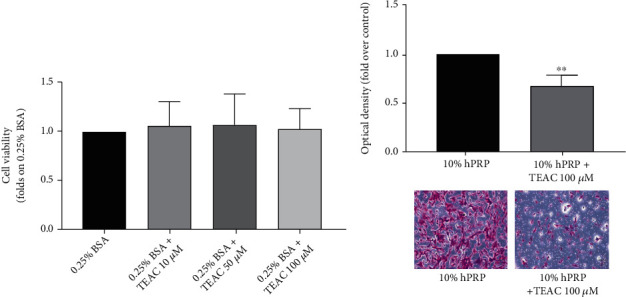
Effect of TEAC inhibitor. (a) cMSCs were treated with three different concentrations of TEAC (10 *μ*M, 50 *μ*M, and 100 *μ*M) in DMEM F12 0.25% BSA medium. Cell viability was analyzed by the sulforodamine assay. Bars show the viability compared to control cells (those cultured in 0.25% BSA). The data obtained represent the mean ± SD of four independent experiments. (b) cMSCs were seeded in the upper chamber of a transwell culture system in the presence of 10% hPRP in the lower chamber with or without 100 *μ*M TEAC. Cells that migrated across the filter were determined by crystal violet staining. Bars show the fold-over control (cells in the presence of 10% hPRP). Before crystal violet elution, cells were photographed (magnification 10x). The data represent the mean ± SD of three independent experiments (^∗^*p* < 0.05).

To prove the direct involvement of AQPs in hPRP-induced cMSC migration, the AQP inhibitor Tetraethylammoniumchloride (TEAC) [25, 30] was used. 10, 50, and 100 *μ*M TEAC treatment did not modify cMSC viability compared to control cells (Figure 5(a)). Interestingly, in the presence of TEAC, 10% hPRP-treated cells displayed a significant reduction of migration compared to control cells (Figure 5(b)).

## 4. Discussion

Mesenchymal stem cells (MSCs) have a clear role in tissue repair after injury due to their ability to migrate into damaged tissues, secrete angiogenic and antiapoptotic factors, and differentiate into specialized cells [31–33]. Moreover, MSCs release a plethora of nano- and microvesicles, such as exosomes, apoptotic bodies, nanoparticles, and microvesicles conveying a multitude of information, and stimuli supporting the regenerative processes [34]. Application of human PRP (hPRP) in human tissue regeneration has also largely been described, as platelets display the ability to release high concentration of molecules involved in the wound healing process [10, 35]. Similar to humans, regenerative capacity of canine adipose-derived MSCs (cMSCs) has been demonstrated [36, 37], highlighting the high potential of these cells in regenerative medicine approaches for dogs.

Efficacy of canine PRP (cPRP) in regenerative medicine has also been demonstrated. In particular, cPRP has provided a beneficial effect for canine skin ulcers in clinical practice, even though some contradictory results have been shown related to different experimental wound models, PRP preparation techniques, and time-point investigations [15, 38]. It has been recently demonstrated that topical applications of PRP, repeated twice with a time gap of 2 weeks, lead to complete closure and epithelialization of large subacute skin lesions in dogs [15].

Here, we have defined the effect of hPRP on cMSC proliferation and migration and the potential use of hPRP for the treatment of small size dog wounds.

Dogs are classified according to breed size. Small-size dogs are represented by animals with a weight of <10-15 kg [39]. Blood sample collection to obtain cPRP is limited by the small quantity of blood that it is possible to obtain from small dogs with a low weight. Thus, the possibility of collecting adequate quantities of cPRP from small dogs for regenerative tissue applications and repair of damaged tissues after large wounds or major burns is limited, especially when more applications are required. Therefore, the advantage of using hPRP consists of acquiring the needed quantities of PRP from human blood samples.

Here, we have characterized cMSCs and defined the effect of hPRP on cMSC proliferation and migration and the potential use of hPRP for the treatment of small-size dog wounds. In agreement with a previous work [40], we have observed that isolated cMSC-stained positive for CD90, CD44, and CD105, standardized markers of mesenchymal stem features for human cells [41], and retained the ability to differentiate both in adipocytes and in osteocyte-like cells. However, as also demonstrated by Rashid et al. [40], in dogs, subcutaneous adipose tissue-derived MSCs do not display a great adipogenic potential, especially when compared to omental adipose tissue-derived MSCs. Next, we assessed the hPRP effect, and first, we demonstrated that hPRP did not modify DLA gene expression and promoted proliferation of adipose-derived cMSCs. This result is in agreement with other studies based on the evaluation of cPRP on cMSC proliferation [38, 42, 43]. However, in these few previous studies, different cPRP procedures and cMSC sources were used. Interestingly, we showed that hPRP had an incremental effect on cMSC migration. According to the recent literature, molecular mechanisms sustaining cMSC proliferation and migration involve ERK1/2, AKT/PKB, and FAK activation induced by hPRP ([8, 44]; suppl. Figure 2). Interestingly, here, we provided evidence that hPRP also induced expression of Aquaporins (AQP) 1 and -5 in cMSCs. Aquaporin inhibition counteracted hPRP-induced cMSC migration (Figure 5). AQP deficiency has been correlated with a reduction in intracellular ATP amount and an alteration of the mitogen-activated protein kinases (MAPK) signaling pathway in epidermal cells [45]. AQPs are water channels localized on extracellular membranes where they could regulate osmotic water flow across the plasma membrane. A crucial role for AQP in the regulation and support of cellular migration has been described in angiogenesis, tumor metastasis, wound healing, glial scarring, and other events requiring rapid, directed cell movement [25, 46]. In particular, AQP5 is likely to play an important role in the migration of human breast cancer cells and in nonsmall cancer lung cells (NSCLC) [26, 27]. AQP1 has also been shown to be involved in erythropoietin-induced endothelial cell migration [28]. Further evidence suggested an AQP-dependent migration mechanism based on actin depolymerization and ion influx increase, who lead to the increase of lamellipodial dynamics to the leading edge of migrating cells [47]. AQP deficiency in a mouse model reduced cutaneous wound healing and compromised epithelial cell regeneration in mouse colon tissues [45], corroborating the role of AQP in sustaining adMSC migration.

Thus, we have shown for the first time that hPRP increases AQP cellular contents and enhances the migration and proliferation of cMSCs. AQP function clearly contributes to cMSC migration but not proliferation. Moreover, we have provided evidence of a helpful effect of hPRP in canine wound healing, highlighting the potential effect of hPRP on tissue-resident cMSCs and opening the scenario to the combined human PRP–canine MSC therapy. Thus, the possible species-heterogeneity limit is overcome by the evidence that human-derived PRP increases migration and proliferation of adMSC from canine species, that is crucial for wound closure. However, it should be noticed that the work is based on *in vitro* observations; *in vivo* studies will be needed to validate the results. For instance, *in vivo* studies will better clarify the immune response of dogs to hPRP since cMSCs are not immune cells and the evaluation of DLA gene expression may not be a sufficient parameter for determining hPRP immunogenicity. In addition, to the best of our knowledge, no *in vitro* studies have described molecules able to increase expression of primary class of major histocompatibility complex I genes, DLA12 and DLA64, on canine MSCs.

Moreover, additional studies are needed to compare the effects of hPRP and cPRP in both *in vitro* and *in vivo* models. Until now, multiple commercial PRP separation systems have been developed and validated for human use. These systems display high variability, giving PRP with different concentrations of platelets, white blood cells, and growth factors. Of course, the variability can be attributed also to patient- (and species-) related factors. For canine use, there are few studies supporting PRP system validation. In addition, it has been observed that PRP systems validated for humans had not similar or consistent results in dogs [17, 48]. Finally, further research should also investigate the potential issues related to the biomedical applications of PRP for canine wound regeneration. Recently, it has been shown that PRP embedded in hydrogels enhances wound healing and creates an adequate microenvironment for cell migration and proliferation [49]. Thus, it is conceivable that PRP could be incorporated in novel hydrogel-based biomedical devices as recently it has been described for two new promising biomaterials: phosphorene and borophene [50, 51]. Further works will be needed to compare these different approaches.

## Data Availability

The data that support the findings of this study are available from the corresponding author upon reasonable request.

## Abbreviations

PRP: Platelet-rich plasma
hPRP: Human platelet-rich plasma
MSC: Mesenchymal stem cells
adMSC: Adipose derived-mesenchymal stem cells
cMSC: Canine mesenchymal stem cells
AQP: Aquaporin
PDGF: Platelet-derived growth factor
TGF-*β*: Transforming growth factors *β*
IGF-1: Insulin-like growth factor-1
FGF: Fibroblast growth factor
FBS: Fetal bovine serum
CM: Conditioned media
BSA: Bovine serum albumin
TEAC: Tetraethylammonium chloride
PBS: Phosphate-buffered saline
MHC-I: Major histocompatibility complex I
DLA: Dog leucocyte antigen
GAPDH: Glyceraldehyde-3-phosphate dehydrogenase
ECL: Enhanced Chemiluminescence
ATP: Adenosine triphosphate
MAPK: mitogen-activated protein kinases
AKT/PKB: Protein kinase B
ERK: Extracellular signal-regulated kinase
FAK: Focal adesion kinase
NSCLC: Nonsmall cancer lung cells.

## Supplementary Materials

Supplementary Materials Supplementary Figure 1: hPRP CM effect on cMSC migration. cMSCs were serum-starved for 18 h and then seeded in the upper chamber of a transwell culture system. 10%hPRP-CM (obtained from hPRP gel in 0.25% BSA medium, as described in Material and Methods) was added in the lower chamber for 48 h. As a control, DMEM F12 (1 : 1) with 0.25% BSA was added to the lower compartment. Cells that migrated across the filter were determined by crystal violet, staining as described in Materials and Methods. Bars show the fold-over control represented by cells in 0.25% BSA. Before crystal violet elution, cells were photographed (magnification 10x). ^∗^ denotes statistical significance versus cells in 0.25% BSA medium (^∗^*p* < 0.05). Supplementary Figure 2: hPRP effect on activation of signaling pathways in cMSCs. cMSCs were treated with 10% hPRP-conditioned medium for 24 h. Cell lysates (20 *μ*g protein/sample) were blotted with pERK Thr202/Tyr204, pAKT S473, and pFAK S910 antibodies. To ensure equal protein transfer, membranes were blotted with vinculin antibodies. Filters were revealed by ECL and autoradiography. The autoradiographs shown are representative of three independent experiments. Densitometric analysis has been performed on autoradiographs. Bars show the ratio between posphoproteins and vinculin protein levels. The data in the graphs represent the mean ± SD of three experiments (^∗^*p* < 0.05).
