# Supplementary material for: Human Platelet-Rich Plasma Regulates Canine Mesenchymal Stem Cell Migration through Aquaporins
Source: Stem Cells Int. 2023 May 15;2023:8344259. doi: 10.1155/2023/8344259 (PMC10202607; doi:10.1155/2023/8344259)
Supplement: Supplementary Materials — Supplementary Figure 1: hPRP CM effect on cMSC migration. cMSCs were serum-starved for 18 h and then seeded in the upper chamber of a transwell culture system. 10%hPRP-CM (obtained from hPRP gel in 0.25% BSA medium, as described in Material and Methods) was added in the lower chamber for 48 h. As a control, DMEM F12 (1 : 1) with 0.25% BSA was added to the lower compartment. Cells that migrated across the filter were determined by crystal violet, staining as described in Materials and Methods. Bars show the fold-over control represented by cells in 0.25% BSA. Before crystal violet elution, cells were photographed (magnification 10x). ∗ denotes statistical significance versus cells in 0.25% BSA medium (∗p < 0.05). Supplementary Figure 2: hPRP effect on activation of signaling pathways in cMSCs. cMSCs were treated with 10% hPRP-conditioned medium for 24 h. Cell lysates (20 μg protein/sample) were blotted with pERK Thr202/Tyr204, pAKT S473, and pFAK S910 antibodies. To ensure equal protein transfer, membranes were blotted with vinculin antibodies. Filters were revealed by ECL and autoradiography. The autoradiographs shown are representative of three independent experiments. Densitometric analysis has been performed on autoradiographs. Bars show the ratio between posphoproteins and vinculin protein levels. The data in the graphs represent the mean ± SD of three experiments (∗p < 0.05). [file 8344259.f1.docx]

SUPPORTING INFORMATION


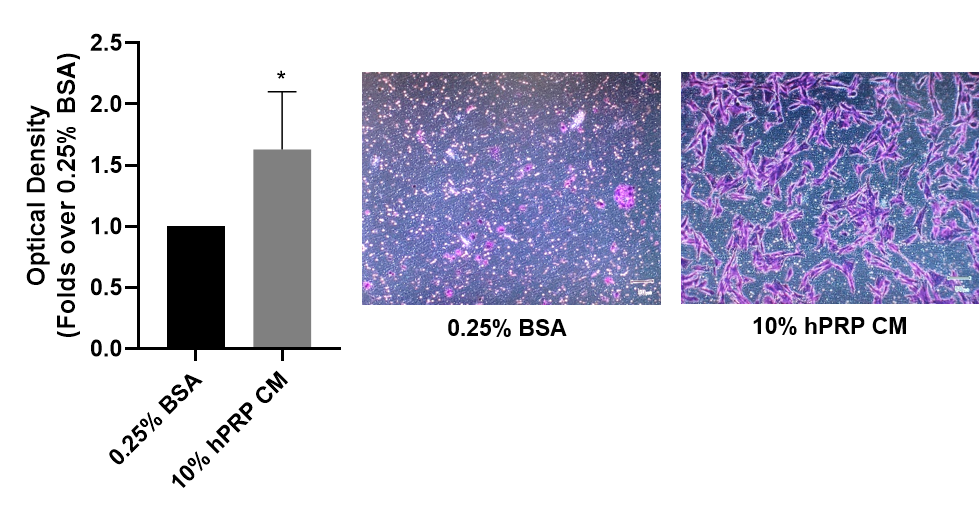


**Supplementary Figure 1: hPRP CM effect on cMSC migration.** cMSCs were serum starved for 18h and then seeded in the upper chamber of a transwell culture system. 10%hPRP-CM (obtained from hPRP gel in 0.25% BSA medium, as described in Material and Methods) was added in the lower chamber for 48h. As control, DMEM F12 (1:1) 0.25% BSA was added to the lower compartment. Cells that migrated across the filter were determined by crystal violet staining as described in Materials and Methods. Bars show the fold over control represented by cells in 0.25% BSA. Before crystal violet elution, cells were photographed (magnification 10X). *denotes statistical significance versus cells in 0.25% BSA medium (*p<0.05).


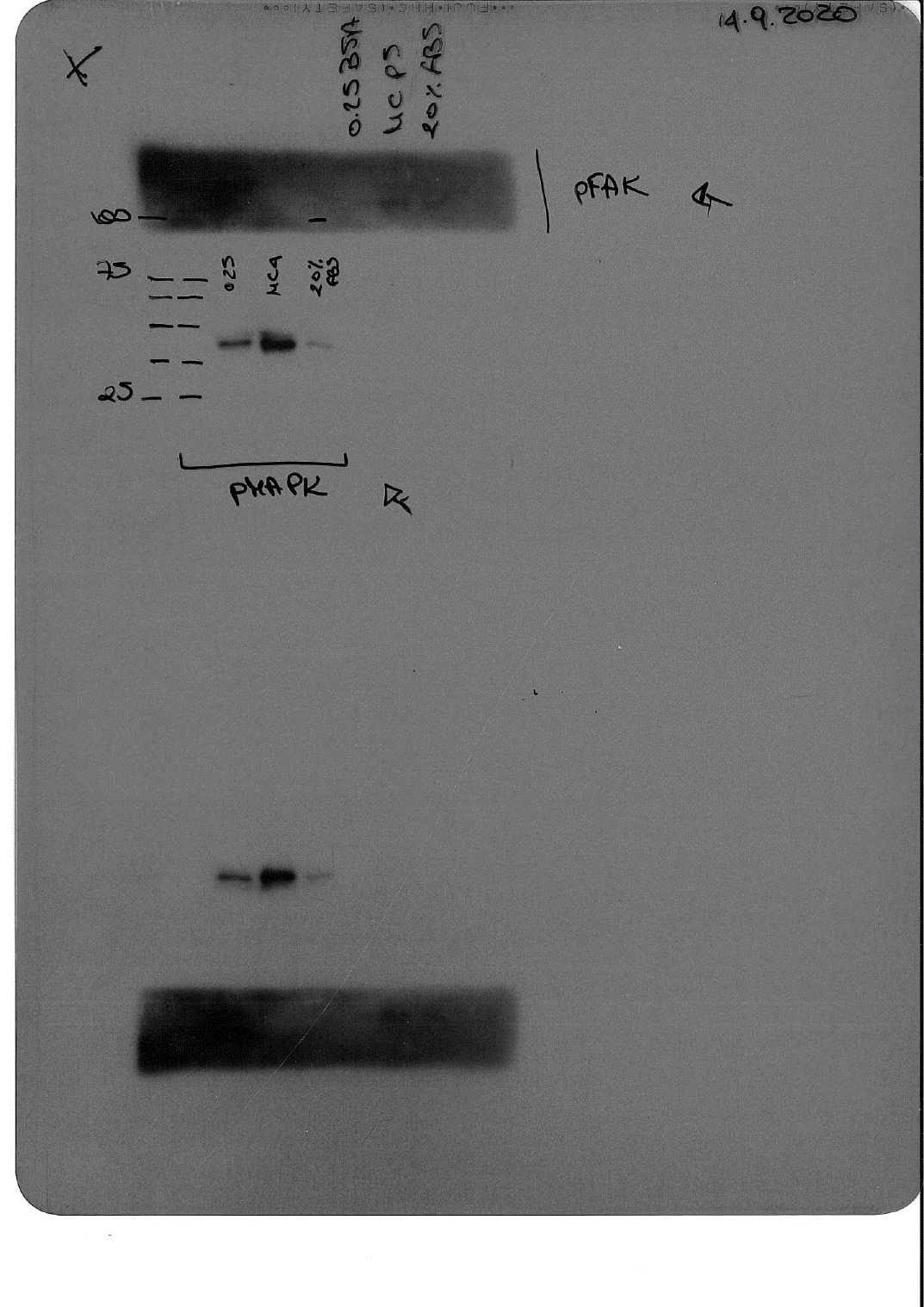


**P-ERK1/2**


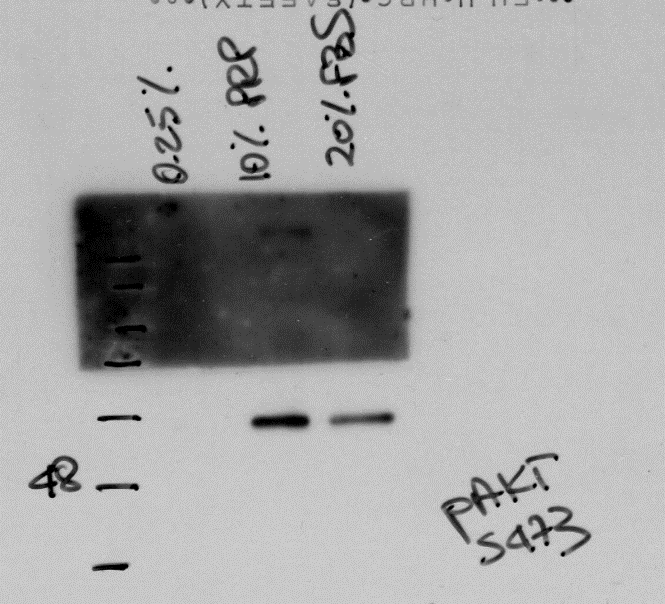


**P-AKT S473**


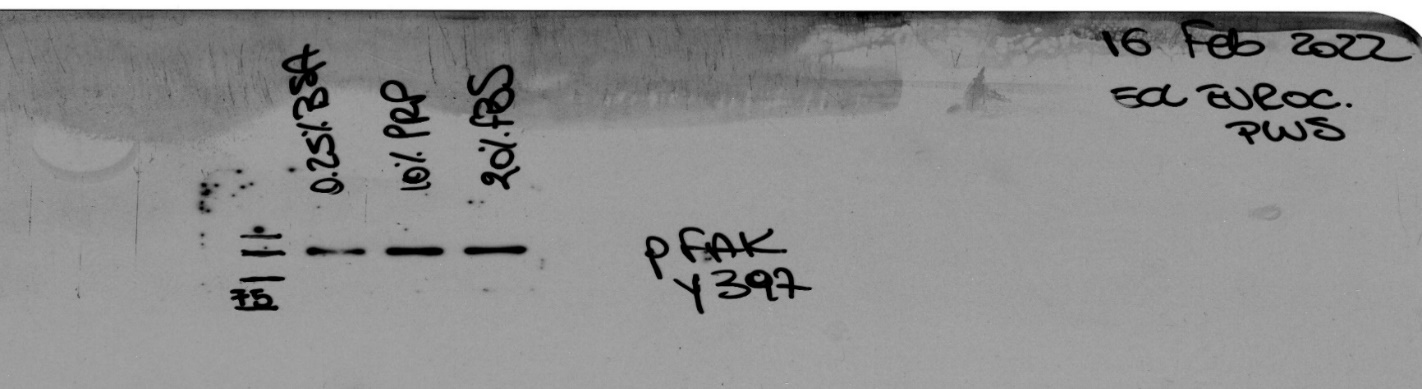


**P-FAK**

**VINCULIN**

**100 -**

**35 -**

**63 -**

**100 -**

**0.25%BSA**

**10%hPRP**


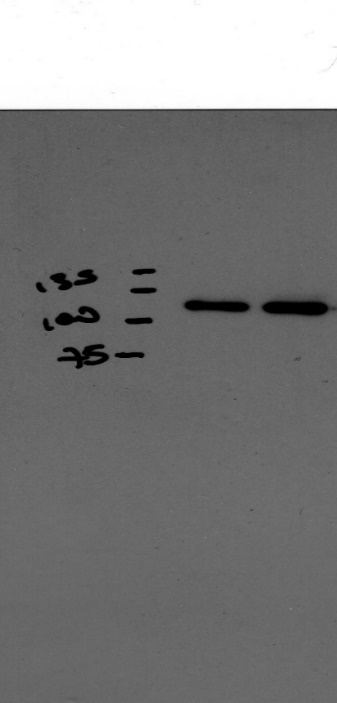


**Supplementary Figure 2: hPRP effect on activation of signaling pathways in cMSCs.** cMSCs were treated with 10%hPRP conditioned medium for 24h. Cell lysates (20 μg protein/sample) were blotted with pERK Thr202/Tyr204, pAKT S473 and pFAK S910 antibodies. To ensure the equal protein transfer, membranes were blotted with vinculin antibodies. Filters were revealed by ECL and autoradiography. The autoradiographs shown are representative of three independent experiments. Densitometric analysis has been performed on autoradiographs. Bars show the ratio between pospho proteins and vinculin protein levels. Data in the graphs represent the mean ± SD of three experiments (*p<0.05).
